# Two-wave panel survey dataset on who feels affected by Hurricane Florence

**DOI:** 10.1016/j.dib.2020.106361

**Published:** 2020-09-29

**Authors:** Talbot M. Andrews, Oleg Smirnov

**Affiliations:** Department of Political Science, Stony Brook University, Center for Behavioral Political Economy, 100 Nicolls Rd, Stony Brook, NY 11794 USA

**Keywords:** Public opinion, Survey, Climate change, Disaster, Hurricane, Empathy, Perspective taking, Helping

## Abstract

Feeling affected by climate change related natural disasters is an important predictor of engaging in climate change mitigation behavior. We therefore collected data to identify who felt affected by Hurricane Florence, which made landfall in the United States on September 14^th^, 2018. In the months before Hurricane Florence, we collected survey responses from a nationally representative sample of United States citizens. We measured their attitudes towards climate change, emotional predispositions, and demographic information. Then, in the days after the hurricane, we re-contacted respondents to identify whether or not they felt personally affected by Hurricane Florence. These data can be used first to identify variables associated with climate change attitudes, and second to identify the traits that predispose individuals to feel affected by climate change related disasters.

## Specifications Table

**Table:** 

Subject	Social Sciences (General)
Specific subject area	Public Opinion
Type of data	Text file Stata data file Stata .do file
How data were acquired	Data was acquired through online questionnaires delivered by YouGov.
Data format	Raw
Parameters for data collection	Respondents were recruited from YouGov's United States panel. The total panel includes over 2 million people in the United States.
Description of data collection	Wave I of the survey was fielded between May 04, 2018 and May 15, 2018. We collected respondent's opinions about climate change, as well as their emotional predispositions. Respondents were then re-contacted for the second wave of the study. The second wave was fielded between September 21, 2018 and September 28, 2018, directly after Hurricane Florence dissipated on September 18^th^, 2018. Respondents were asked whether they had been personally affected by Hurricane Florence. YouGov interviewed a total of 2,244 respondents in Wave 1 who were then matched down to a sample of 1,500 to produce the final dataset. The respondents were matched to a sampling frame on gender, age, race, and education.
Data source location	Country: The United States of America
Data accessibility	Data are available with this article.
Related research article	[1] T.M. Andrews, O. Smirnov, Who feels the impacts of climate change?, Glob. Environ. Chang. (2020). https://doi.org/10.1016/j.gloenvcha.2020.102164

## Value of the Data

• These data identify the characteristics, measured before Hurricane Florence, that predispose individuals to feel affected by the disaster.
• Because feeling affected by climate change related natural disasters is an important predictor of climate change mitigation behavior, these data are useful to those studying how to best mobilize mitigation.
• These data could be further used to identify sensitivity to climate change related disasters.
• Additionally, these data can be used to study the socio-demographic and geographic characteristics that are associated with belief in and concern about climate change.

## 1 Data Description

The data set includes the raw data from both Wave 1 and Wave 2 of our survey. Wave 1 includes responses for all 2,244 respondents who completed the first wave of the study, while the final panel in includes those who completed both waves of the study, with some respondents removed by YouGov to ensure our final panel included a nationally representative 1,500-person panel.

Additionally, the *pdf*-file “Codebook” describes each of the variables and response options. Finally, the do-file includes the Stata code required to generate each of the scales included in the data, as well as the scree plots and factor analyses included below.

**Fig. 1:**
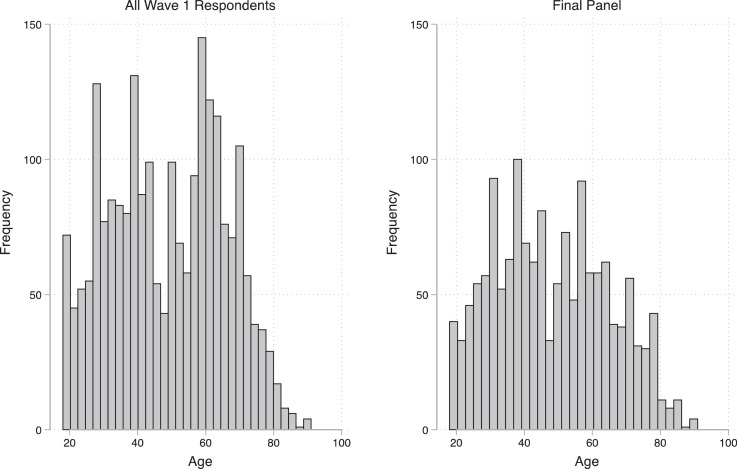
Distribution of respondent ages.

Respondents were recruited for the first wave of the panel through YouGov, a company which maintains an online panel of over 2 million respondents in the United States. Prior to completing either wave, YouGov collected each respondent's socio-demographic information, their geographic location, and their political preferences. Fig. 1 shows the distribution of respondents’ ages, and Table 1, Table 2, Table 3, Table 4, Table 5, Table 6 show the distribution of respondent's demographic and political characteristics.

**Table 1:** Respondent gender.

	Wave 1	Wave 1	Panel	Panel
	Freq.	Percent	Freq.	Percent
Male	1,020	45.5	693	46.2
Female	1,224	54.6	807	53.8
Total	2,244	100	1,500	100

**Table 2:** Respondent race.

	Wave 1	Wave 1	Panel	Panel
	Freq.	Percent	Freq.	Percent
White	1,655	73.75	1,074	71.6
Black	215	9.58	160	10.67
Hispanic	225	10.03	156	10.4
Asian	59	2.63	47	3.13
Native American	17	0.76	13	0.87
Mixed	35	1.56	26	1.73
Other	33	1.47	22	1.47
Middle Eastern	5	0.22	2	0.13
Total	2,244	100	1,500	100

**Table 3:** Respondent education.

	Wave 1	Wave 1	Panel	Panel
	Freq.	Percent	Freq.	Percent
No HS	183	8.16	108	7.2
High school graduate	758	33.78	515	34.33
Some college	435	19.39	294	19.6
2-year	255	11.36	173	11.53
4-year	392	17.47	263	17.53
Post-grad	221	9.85	147	9.8
Total	2,244	100	1,500	100

**Table 4:** Respondent marital status.

	Wave 1	Wave 1	Panel	Panel
	Freq.	Percent	Freq.	Percent
Married	1,095	48.8	709	47.27
Separated	35	1.56	22	1.47
Divorced	237	10.56	150	10
Widowed	115	5.12	79	5.27
Never married	682	30.39	490	32.67
Domestic / civil partnership	80	3.57	50	3.33
Total	2,244	100	1,500	100

**Table 5:** Respondent religion.

	Wave 1	Wave 1	Panel	Panel
Religion	Freq.	Percent	Freq.	Percent
Protestant	797	35.52	523	34.87
Roman Catholic	423	18.85	284	18.93
Mormon	31	1.38	21	1.4
Eastern or Greek Orthodox	17	0.76	9	0.6
Jewish	47	2.09	29	1.93
Muslim	21	0.94	10	0.67
Buddhist	25	1.11	17	1.13
Hindu	6	0.27	5	0.33
Atheist	140	6.24	108	7.2
Agnostic	124	5.53	80	5.33
Nothing in particular	505	22.5	335	22.33
Something else	108	4.81	79	5.27
Total	2,244	100	1,500	100

**Table 6:** Respondent partisan identification.

	Wave 1	Wave 1	Panel	Panel
	Freq.	Percent	Freq.	Percent
Democrat	755	33.65	508	33.87
Republican	535	23.84	351	23.4
Independent	677	30.17	445	29.67
Other	114	5.08	86	5.73
Not sure	163	7.26	110	7.33
Total	2,244	100	1,500	100

In the first wave of the panel we asked respondents about their attitudes surrounding climate change, including whether or not they believe in anthropogenic climate change, as well as how worried they are about climate change and how often they discuss the issue. A total of 2,244 respondents completed the first wave. The full question wording as well as distribution of the responses in the first wave of the data are presented in Table 7.

**Table 7:** Full question wording for each climate change attitude question is presented in the first column. The second column shows each response option, as well as the number of respondents who selected each option out of the total number who completed the first wave of the study (n = 2,244).

Question	Response Options
Do you think that global warming is happening?	Yes: 1,399 No: 511 Don't Know: 334
Assuming global warming is happening, do you think it is…?	Caused mostly by human activities: 1,166 Caused mostly by natural changes in the environment: 660 Other: 128 None of the above because global warming isn't happening: 290
How worried are you about global warming?	Very worried: 620 Somewhat worried: 711 Not very worried: 438 Not at all worried: 475
How often do you discuss global warming with your friends and family?	Often: 223 Occasionally: 791 Rarely: 700 Never: 530

**Fig. 2:**
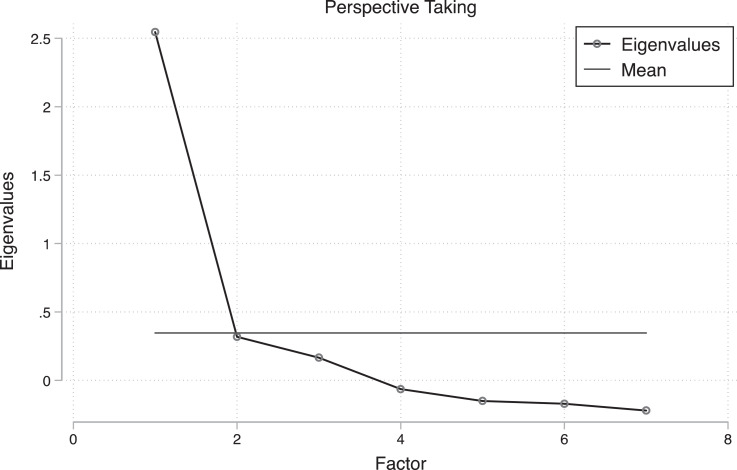
Scree plot for perspective taking scale items.

In the first wave, we additionally ascertained each respondent's emotional predispositions. First, we measure perspective taking abilities, or the propensity to automatically adopt the perspective of others [2], [3], [4]. Each question in the perspective taking scale, as well as a principal factor analysis of the scale, is presented in Table 8. Additionally, the scree plot for the scale, suggesting the scale captures a single dimension, is presented in Fig. 2.

**Table 8:** Principal factor analysis for the perspective taking scale. Scale reliability coefficient Cronbach's alpha = 0.772.

	Factor 1	Factor 2
I sometimes find it difficult to see things from the "other guy's" point of view.	0.347	0.370
I try to look at everybody's side of a disagreement before I make a decision.	0.673	0.047
I sometimes try to understand my friends better by imagining how things look from their perspective.	0.696	-0.062
If I'm sure I'm right about something, I don't waste much time listening to other people's arguments.	0.322	0.345
I believe that there are two sides to every question and try to look at them both.	0.633	0.010
When I'm upset at someone, I usually try to "put myself in his shoes" for a while.	0.683	-0.172
Before criticizing somebody, I try to imagine how I would feel if I were in their place.	0.720	-0.163

**Fig. 3:**
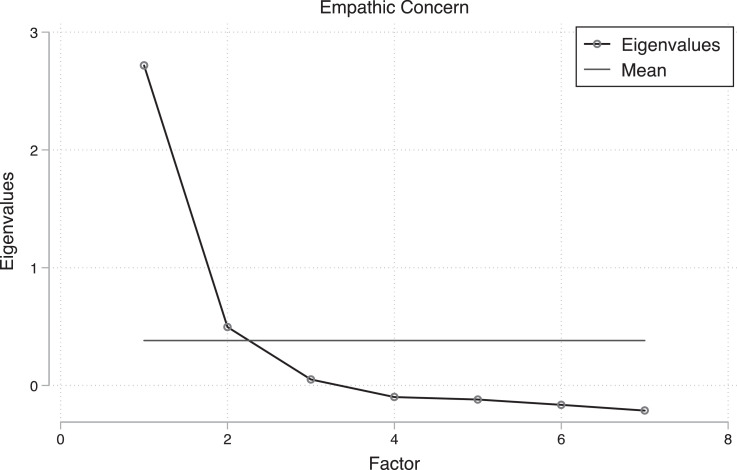
Scree plot for empathic concern scale items.

Second, we measured levels of empathic concern. Though empathic concern is similar to perspective taking in that it is an emotional response to the affective state of another person, it is different in that it is subject to conscious regulation [3], [5], [6], [7]. The items of the empathic concern scale, as well as a principal factor analysis, are in Table 9. Fig. 3 shows the scree plot for the empathic concern scale

**Table 9:** Principal factor analysis for the empathic concern scale. Scale reliability coefficient Cronbach's alpha = 0.806.

	Factor 1	Factor 2
I often have tender, concerned feelings for people less fortunate than me.	0.723	-0.149
Sometimes I don't feel very sorry for other people when they are having problems.	0.486	0.318
When I see someone being taken advantage of, I feel kind of protective towards them.	0.664	-0.177
Other people's misfortunes do not usually disturb me a great deal.	0.633	0.316
When I see someone being treated unfairly, I sometimes don't feel very much pity for them.	0.541	0.348
I am often quite touched by things that I see happen.	0.694	-0.267
I would describe myself as a pretty soft-hearted person.	0.587	-0.226

**Fig. 4:**
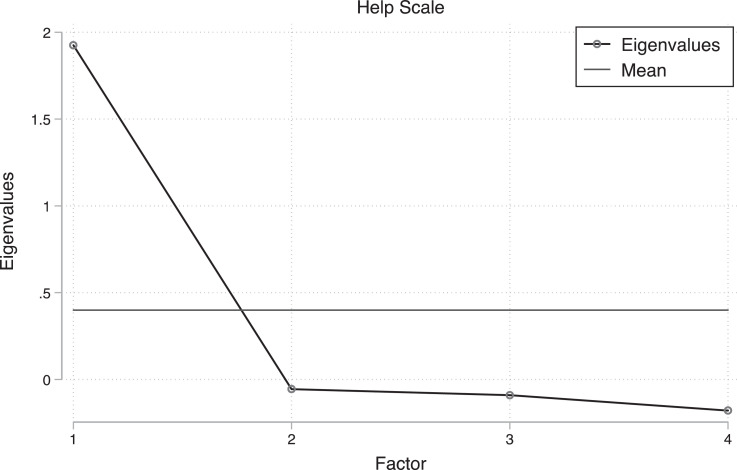
Scree plot for help scale items.

Finally, we constructed a novel scale to measure respondent's willingness to help victims of a hurricane. The scale items and principal factor analysis are presented in Table 10, and the scree plot is presented in Fig. 4.

**Table 10:** Principal factor analysis for the help scale. Scale reliability coefficient Cronbach's alpha = 0.787.

	Factor 1
How likely would you help victims of a hurricane?	0.632
How likely would you assist victims of a hurricane by donating material items such as food and clothes?	0.803
How likely would you assist victims of a hurricane by donating money?	0.677
How likely would you assist victims of a hurricane by volunteering?	0.650

In the second wave of the panel, we re-contacted respondents and asked them whether they were personally affected by Hurricane Florence. In total, 1,500 respondents completed both the first and second wave of the panel. Hurricane Florence made landfall in the United States on September 14^th^, 2018 and primarily impacted North Carolina, South Carolina, and Virginia [8]. Table 11 shows how many respondents completed the second wave of the study in each state, as well as the number of respondents who stated they were personally affected by Hurricane Florence.

**Table 11:** Geographic distribution of survey respondents and who felt affected by Hurricane Florence. The table lists first the total number of respondents from each state in the dataset, and then the total number who report being personally affected by Hurricane Florence. The results only include those who completed both Wave 1 and Wave 2 of the study.

State	Total	Aff.	State	Total	Aff.	State	Total	Aff.
Alabama	23	1	Kentucky	26	6	North Dakota	2	0
Alaska	1	1	Louisiana	12	1	Ohio	65	9
Arizona	36	0	Maine	12	0	Oklahoma	11	0
Arkansas	17	3	Maryland	26	8	Oregon	34	1
California	114	10	Massachusetts	36	3	Pennsylvania	92	10
Colorado	35	4	Michigan	53	3	Rhode Island	1	0
Connecticut	19	0	Minnesota	25	0	South Carolina	33	20
Delaware	8	1	Mississippi	11	3	South Dakota	6	1
D.C.	4	2	Missouri	29	3	Tennessee	32	5
Florida	79	9	Montana	8	1	Texas	32	7
Georgia	39	9	Nebraska	5	0	Utah	5	0
Hawaii	6	0	Nevada	19	0	Vermont	6	0
Idaho	9	0	New Hampshire	9	1	Virginia	36	14
Illinois	69	2	New Jersey	43	5	Washington	39	5
Indiana	34	2	New Mexico	12	1	West Virginia	9	0
Iowa	23	2	New York	97	10	Wisconsin	40	2
Kansas	16	0	North Carolina	46	37	Wyoming	3	1

A detailed description of all other variables included in the questionnaires are available in the codebook in the supplementary files of this data article. Respondent's responses are included in the Stata file, and the included do-file has the code necessary to construct each of the scales in these data.

## 2 Experimental Design, Materials and Methods

Respondents were recruited for the first wave of the study through YouGov, a company which maintains an online panel of over 2 million respondents in the United States. Prior to completing either wave of our study, YouGov collected each respondent's socio-demographic information, their geographic location, and their political preferences.

Wave I was fielded between May 04, 2018 and May 15, 2018, before hurricane season. After agreeing to participate, respondents were randomly assigned to one of three experimental conditions. In the control condition, respondents read a brief paragraph about damages caused by Hurricane Harvey, which hit the United States in the fall of 2017. In the certain attribution condition, respondents additionally read a short paragraph about how climate change is increasing the severity of hurricanes like Hurricane Harvey. In the uncertain attribution condition, respondents read about the damages caused by Hurricane Harvey and then read a short paragraph about how it is unclear if climate change exacerbated those damages. Respondents then answered questions about their climate change attitudes and emotional predispositions.

For the second wave of the study, YouGov re-contacted those who had completed Wave I. The final sample of 1,500 included all respondents who completed both waves of the study, and then was matched down to a sampling frame on gender, age, race, and education. While this reduced the total size of our final data set, it ensures the data is as representative of the U.S. population as possible. Wave II was fielded from September 21, 2018 to September 28, 2018, after Hurricane Florence dissipated on September 18^th^.

After agreeing to participate, respondents were again randomly assigned to one of three experimental conditions. In all three conditions, respondents read a short paragraph about the damages caused by Hurricane Florence. In the certain attribution condition, respondents additionally read about how these damages were exacerbated by climate change. In the uncertain attribution condition, respondents read about the hurricane damages, as well as how it was uncertain whether climate change contributed to the severity of the storm. All respondents then reported whether they had been personally affected by the hurricane.

All survey questions and text from the experiments are included in the codebook in the supplemental information included with this paper.

## Ethics Statement

Informed consent was obtained from all participants before their participation in this study. The work was conducted with the approval of the authors’ Institutional Review Board.

## Declaration of Competing Interest

The authors declare that they have no known competing financial interests or personal relationships which have, or could be perceived to have, influenced the work reported in this article.

## Appendix Supplementary materials

Image, application 1
