# Supplementary material for: Two-wave panel survey dataset on who feels affected by Hurricane Florence
Source: Data Brief. 2020 Sep 29;33:106361. doi: 10.1016/j.dib.2020.106361 (PMC7548421; doi:10.1016/j.dib.2020.106361)
Supplement: Supplementary file 1 [file mmc1.zip › Supplementary Material/Codebook.pdf]

Codebook for:

Two-wave panel survey dataset on who feels affected by Hurricane Florence

Contents:

1. Wave 1 Treatments and Questionnaire
2. Wave 2 Treatments and Questionnaire
3. Demographic information collected by YouGov

Wave 1 Questionnaire

We are conducting a survey regarding public reactions to hurricanes. This survey will take approximately 15 minutes. All your answers will be kept confidential. You do not have to participate in this study if you do not want to. For your information, the study is funded by the National Science Foundation. With your permission, let's begin.

INTRODUCTION. PLEASE READ CAREFULLY:
Hurricane Harvey was the first major hurricane to make landfall in the United States since Wilma in 2005, ending a record 12-year drought in which no hurricanes made landfall at such an intensity in the country. Harvey caused at least 71 confirmed deaths; 1 in Guyana, and 70 in the United States. FEMA director Brock Long called Harvey the worst disaster in Texas history, and expected the recovery to take many years. Economic losses are preliminarily estimated at between $70 to $200 billion, with a large portion of the losses sustained by uninsured homeowners.

(RANDOMIZE CONDITIONS 1-3)
CONDITION #1: (EMPTY)

CONDITION #2:
Did global warming cause or have any impact on the 2017 hurricanes? Scientists are uncertain about whether global warming will cause more hurricanes. In fact, some scientists say that a warmer planet will actually result in fewer hurricanes. It's worth noting that there are other things that made Irma big that have no clear association with climate change. Vertical wind shear in the hurricane "nursery" region of the Atlantic are weak this year. Both Hurricane Harvey and Hurricane Irma could have developed, regardless of the warming climate. According to University of Washington atmospheric scientist Cliff Mass: "The bottom line in this analysis is that both observations of the past decades and models looking forward to the future do not suggest that one can explain the heavy rains of Harvey by global warming, and folks that are suggesting it are poorly informing the public and decision makers."

CONDITION #3:
Did global warming cause or have any impact on the 2017 hurricanes? "It is not a coincidence that we're seeing more devastating hurricanes," says climatologist Michael Mann of Penn State University. "Over the past few years, as global sea surface temperatures have been the warmest on record, we've seen the strongest hurricanes... The impacts of climate change are no longer subtle... The science is now fairly clear that climate change will make stronger storms stronger." Climate scientist Kevin Trenberth from the National Center for Atmospheric Research explains: "Previous very active (hurricane) years were 2005 and 2010," he says, and along with 2017, they experienced warm Atlantic Ocean temperatures. "So this sets the stage. So the overall trend is global warming from human activities."

[past_hurr1] Have you ever been personally affected by hurricanes in the past?

1. Affected a great deal
2. Somewhat affected
3. Not affected

[past_hurr2] Have any member of your family or close friends been personally affected by hurricanes in the past?

1. Affected a great deal
2. Somewhat affected
3. Not affected

[help1] How likely would you help victims of a hurricane?

1. Extremely Likely
2. Very Likely
3. Moderately Likely
4. Slightly Likely
5. Neither Likely not Unlikely
6. Slightly Unlikely
7. Moderately Unlikely
8. Very Unlikely
9. Extremely Unlikely

[help2] How likely would you assist victims of a hurricane by donating material items such as food and clothes?

1. Extremely Likely
2. Very Likely
3. Moderately Likely
4. Slightly Likely
5. Neither Likely not Unlikely
6. Slightly Unlikely
7. Moderately Unlikely
8. Very Unlikely
9. Extremely Unlikely

[help3] How likely would you assist victims of a hurricane by donating money?

1. Extremely Likely
2. Very Likely
3. Moderately Likely
4. Slightly Likely
5. Neither Likely not Unlikely
6. Slightly Unlikely
7. Moderately Unlikely
8. Very Unlikely
9. Extremely Unlikely

[help4] How likely would you assist victims of a hurricane by volunteering?

1. Extremely Likely
2. Very Likely
3. Moderately Likely
4. Slightly Likely
5. Neither Likely not Unlikely
6. Slightly Unlikely
7. Moderately Unlikely
8. Very Unlikely
9. Extremely Unlikely

Global warming refers to the idea that the world's average temperature has been increasing over the past 150 years, may be increasing more in the future, and that the world's climate may change as a result. What do you think:

[gw_real] Do you think that global warming is happening?
1. Yes
2. No
3. Don't know

[gw_anthro] Assuming global warming is happening, do you think it is… ?
1. Caused mostly by human activities
2. Caused mostly by natural changes in the environment
3. Other
4. None of the above because global warming isn't happening

[gw_worry] How worried are you about global warming?
1. Very worried
2. Somewhat worried
3. Not very worried
4. Not at all worried

[gw_discuss] How often do you discuss global warming with your friends and family?
1. Often
2. Occasionally
3. Rarely
4. Never

The following statements inquire about your thoughts and feelings in a variety of situations. For each item, indicate how well it describes you. Answer as honestly as you can.

[ec1] I often have tender, concerned feelings for people less fortunate than me.
1. Strongly disagree
2. Disagree
3. Neither agree nor disagree
4. Agree
5. Strongly agree

[pt1] I sometimes find it difficult to see things from the "other guy's" point of view.
1. Strongly disagree
2. Disagree
3. Neither agree nor disagree
4. Agree
5. Strongly agree

[ec2] Sometimes I don't feel very sorry for other people when they are having problems.
1. Strongly disagree
2. Disagree
3. Neither agree nor disagree
4. Agree
5. Strongly agree

[pt2] I try to look at everybody's side of a disagreement before I make a decision.
1. Strongly disagree
2. Disagree
3. Neither agree nor disagree
4. Agree
5. Strongly agree

[ec3] When I see someone being taken advantage of, I feel kind of protective towards them.
1. Strongly disagree
2. Disagree
3. Neither agree nor disagree
4. Agree
5. Strongly agree

[pt3] I sometimes try to understand my friends better by imagining how things look from their perspective.
1. Strongly disagree
2. Disagree
3. Neither agree nor disagree
4. Agree
5. Strongly agree

[ec4] Other people's misfortunes do not usually disturb me a great deal.

1. Strongly disagree
2. Disagree
3. Neither agree nor disagree
4. Agree
5. Strongly agree

[pt4] If I'm sure I'm right about something, I don't waste much time listening to other people's arguments.

1. Strongly disagree
2. Disagree
3. Neither agree nor disagree
4. Agree
5. Strongly agree

[ec5] When I see someone being treated unfairly, I sometimes don't feel very much pity for them.

1. Strongly disagree
2. Disagree
3. Neither agree nor disagree
4. Agree
5. Strongly agree

[ec6] I am often quite touched by things that I see happen.

1. Strongly disagree
2. Disagree
3. Neither agree nor disagree
4. Agree
5. Strongly agree

[pt5] I believe that there are two sides to every question and try to look at them both.

1. Strongly disagree
2. Disagree
3. Neither agree nor disagree
4. Agree
5. Strongly agree

Q32. *[ec7]* I would describe myself as a pretty soft-hearted person.

1. Strongly disagree
2. Disagree
3. Neither agree nor disagree
4. Agree
5. Strongly agree

[pt6] When I'm upset at someone, I usually try to "put myself in his shoes" for a while.

1. Strongly disagree
2. Disagree
3. Neither agree nor disagree
4. Agree
5. Strongly agree

Q34. *[pt7]* Before criticizing somebody, I try to imagine how I would feel if I were in their place.

1. Strongly disagree
2. Disagree
3. Neither agree nor disagree
4. Agree
5. Strongly agree

Wave 2 Questionnaire

We are conducting a survey regarding public reactions to hurricanes. This survey will take approximately 15 minutes. All your answers will be kept confidential. You do not have to participate in this study if you do not want to. For your information, the study is funded by the National Science Foundation. With your permission, let's begin.

INTRODUCTION. PLEASE READ CAREFULLY:

Hurricane Florence was the first major hurricane of the 2018 Atlantic hurricane season. Moody's Analytics estimated that property damage from the hurricane may exceed $22 billion, with thousands of homes destroyed by catastrophic flooding. According to CBS News, as of Sept. 21st, Florence killed at least 42 people, including children, in three states: North Carolina, South Carolina, and Virginia.

(RANDOMIZE CONDITIONS 1-3)
CONDITION #1: (EMPTY)

CONDITION #2:
Did global warming cause or have any impact on the 2018 hurricanes? Scientists are uncertain about whether global warming will cause more hurricanes. In fact, some scientists say that a warmer planet will actually result in fewer hurricanes. Florence could have developed regardless of the warming climate. According to University of Washington atmospheric scientist Cliff Mass, “observations of the past decades and models looking forward to the future do not suggest that one can explain the heavy rains of [a major hurricane] by global warming, and folks that are suggesting it are poorly informing the public and decision makers.”

CONDITION #3:
Did global warming cause or have any impact on the 2018 hurricanes? "It is not a coincidence that we're seeing more devastating hurricanes," says climatologist Michael Mann of Penn State University. "Over the past few years, as global sea surface temperatures have been the warmest on record, we've seen the strongest hurricanes... The impacts of climate change are no longer subtle... The science is now fairly clear that climate change will make stronger storms stronger." Climate scientist Kevin Trenberth from the National Center for Atmospheric Research explains: "Previous very active (hurricane) years were 2005 and 2010," he says, and along with 2017, they experienced warm Atlantic Ocean temperatures. "So this sets the stage. So the overall trend is global warming from human activities."

[florence] Were you personally affected by Hurricane Florence?
1. Affected a great deal.
2. Somewhat affected.
3. Not affected.

Demographic information collected by YouGov

[birthyr] Respondent birth year.

[gender] Respondent gender.
1. Male
2. Female

[race] Respondent race.
1. White
2. Black
3. Hispanic
4. Asian
5. Native American
6. Mixed
7. Other
8. Middle Eastern

[educ] Respondent education.
1. No high school
2. High school graduate
3. Some college
4. 2-year degree
5. 4-year degree
6. Post-graduate degree

[marstat] Respondent marital status.
1. Married
2. Separated
3. Divorced
4. Widowed
5. Never married
6. Domestic/Civil partnership

[employ] Respondent employment status.
1. Full-time
2. Part-time
3. Temporarily laid off
4. Unemployed
5. Retired
6. Permanently disabled
7. Homemade
8. Student
9. Other

[faminc_new] Respondent family income.

1. Less than $10,000
2. $10,000 - $19,999
3. $20,000 - $29,999
4. $30,000 - $39,999
5. $40,000 - $49,999
6. $50,000 - $59,999
7. $60,000 - $69,999
8. $70,000 - $79,999
9. $80,000 - $99,999
10. $100,000 - $119,999
11. $120,000 - $149,999
12. $150,000 - $199,999
13. $200,000 - $249,999
14. $250,000 - $349,999
15. $350,000 - $499,999
16. $500,000 or more
17. Prefer not to say

[pid7] Respondent partisanship.

1. Strong Democrat
2. Not very strong Democrat
3. Lean Democrat
4. Independent
5. Lean Republican
6. Not very strong Republican
7. Strong Republican
8. Not sure

[inputstate] State of residence.

[casscounty] County of residence.

[casszip] Respondent zip code.

[votereg] Is the respondent registered to vote?

1. Yes
2. No
3. Don’t know

[turnout16] Did the respondent vote in the 2016 presidential election?

1. Yes
2. No

[presvote16post] Who did the respondent vote for in the 2016 presidential election?

1. Hillary Clinton
2. Donald Trump
3. Gary Johnson
4. Jill Stein
5. Evan McMullin
6. Other
7. Did not vote for President

[ideo5] Respondent ideology.

1. Very liberal
2. Liberal
3. Moderate
4. Conservative
5. Very Conservative
6. Not sure

[religpew] Respondent religion.

1. Protestant
2. Roman Catholic
3. Mormon
4. Eastern or Greek Orthodox
5. Jewish
6. Muslim
7. Buddhist
8. Hindu
9. Atheist
10. Agnostic
11. Nothing in particular
12. Something else

[pew_churatd] Church attendance.

1. More than once a week
2. Once a week
3. Once or twice a month
4. A few times a year
5. Seldom
6. Never
7. Don’t know
