# Supplementary material for: Two-wave panel survey dataset on who feels affected by Hurricane Florence
Source: Data Brief. 2020 Sep 29;33:106361. doi: 10.1016/j.dib.2020.106361 (PMC7548421; doi:10.1016/j.dib.2020.106361)
Supplement: Supplementary file 1 [file mmc1.zip › Supplementary Material/Data in Brief DoFile.rtf]

******************************************************************************************* Two-wave panel survey dataset on who feels affected by Hurricane Florence **Do-File for data cleaning 								      *	*******************************************************************************************~* Data Cleaning *~* *Identifying respondents who lived in states affected by Hurricane Florence*North Carolinagen NC_resident = 0replace NC_resident = 1 if inputstate == 37*South Carolinagen SC_resident = 0replace SC_resident = 1 if inputstate == 45*Virginiagen VA_resident = 0replace VA_resident = 1 if inputstate == 51*Any of the three affected statesgen hit_state = 0replace hit_state = 1 if(NC_resident == 1)replace hit_state = 1 if(SC_resident == 1)replace hit_state = 1 if(VA_resident == 1)*~* Age  *~~~~~~~~~~~~~~~~~~~~~~~~~~~~~~~~~~~~~~~~~~~~~~~~~~~~~~~~~~~~~~~~~~~~~* *Generating age from birthyeargen age = 2018 - birthyr*Distribution of age*~* Empathic Concern Scale  *~~~~~~~~~~~~~~~~~~~~~~~~~~~~~~~~~~~~~~~~~~~~~~~~~~* *Accounting for reverse coding, scaling each response from 0-1gen ec_21 = (5 - ec1)/4gen ec_23 = (ec2 - 1)/4gen ec_25 = (5 - ec3)/4gen ec_27 = (ec4 - 1)/4gen ec_29 = (ec5 - 1)/4gen ec_30 = (5 - ec6)/4gen ec_32 = (5 - ec7)/4*Generating Empathic Concern Scale, & scale standard deviationegen ec_sd = rowsd (ec_21 ec_23 ec_25 ec_27 ec_29 ec_30 ec_32)egen ec_scale = rowmean (ec_21 ec_23 ec_25 ec_27 ec_29 ec_30 ec_32)*Principal Factor Analysisfactor ec_21 ec_23 ec_25 ec_27 ec_29 ec_30 ec_32*Cronbach's Alphaalpha ec_21 ec_23 ec_25 ec_27 ec_29 ec_30 ec_32*Scree plotscreeplot, mean xtitle("Factor") ytitle("Eigenvalues") title("Empathic Concern") ///	legend(ring(0) pos(2) size(med) region(lcolor(black) lpattern(solid)))	*~* Perspective Taking Scale  *~~~~~~~~~~~~~~~~~~~~~~~~~~~~~~~~~~~~~~~~~~~~~~~~* *Accounting for reverse coding, scaling each response from 0-1gen pt_22 = (pt1 - 1)/4gen pt_24 = (5 - pt2)/4gen pt_26 = (5 - pt3)/4gen pt_28 = (pt4 - 1)/4gen pt_31 = (5 - pt5)/4gen pt_33 = (5 - pt6)/4gen pt_34 = (5 - pt7)/4*Generating Perspective Taking Scale, & scale standard deviationegen pt_sd = rowsd (pt_22 pt_24 pt_26 pt_28 pt_31 pt_33 pt_34)egen pt_scale = rowmean (pt_22 pt_24 pt_26 pt_28 pt_31 pt_33 pt_34)*Principal Factor Analysisfactor pt_22 pt_24 pt_26 pt_28 pt_31 pt_33 pt_34*Cronbach's Alphaalpha pt_22 pt_24 pt_26 pt_28 pt_31 pt_33 pt_34*Scree plotscreeplot, mean xtitle("Factor") ytitle("Eigenvalues") title("Perspective Taking") ///	legend(ring(0) pos(2) size(med) region(lcolor(black) lpattern(solid)))*~* Help Scale  *~~~~~~~~~~~~~~~~~~~~~~~~~~~~~~~~~~~~~~~~~~~~~~~~~~~~~~~~~~~~~~* *Accounting for reverse coding, scaling each response from 0-1gen help_q3 = (9 - help1)/8gen help_q4 = (9 - help2)/8gen help_q5 = (9 - help3)/8gen help_q6 = (9 - help4)/8*Generating Help Scaleegen help_scale = rowmean(help_q3 help_q4 help_q5 help_q6)*Principal Factor Analysisfactor help_q3 help_q4 help_q5 help_q6*Cronbach's Alphaalpha help_q3 help_q4 help_q5 help_q6*Scree plotscreeplot, mean xtitle("Factor") ytitle("Eigenvalues") title("Help Scale") ///	legend(ring(0) pos(2) size(med) region(lcolor(black) lpattern(solid)))
